# When Viruses Don’t Go Viral: The Importance of Host Phylogeographic Structure in the Spatial Spread of Arenaviruses

**DOI:** 10.1371/journal.ppat.1006073

**Published:** 2017-01-11

**Authors:** Sophie Gryseels, Stuart J. E. Baird, Benny Borremans, Rhodes Makundi, Herwig Leirs, Joëlle Goüy de Bellocq

**Affiliations:** 1 Evolutionary Ecology Group, Department of Biology, University of Antwerp, Antwerp, Belgium; 2 Institute of Vertebrate Biology of the Czech Academy of Sciences, Research Facility Studenec, Brno, Czech Republic; 3 Pest Management Centre, Sokoine University of Agriculture, Morogoro, Tanzania; Division of Clinical Research, UNITED STATES

## Abstract

Many emerging infections are RNA virus spillovers from animal reservoirs. Reservoir identification is necessary for predicting the geographic extent of infection risk, but rarely are taxonomic levels below the animal species considered as reservoir, and only key circumstances in nature and methodology allow intrinsic virus-host associations to be distinguished from simple geographic (co-)isolation. We sampled and genetically characterized in detail a contact zone of two subtaxa of the rodent *Mastomys natalensis* in Tanzania. We find two distinct arenaviruses, Gairo and Morogoro virus, each spatially confined to a single *M*. *natalensis* subtaxon, only co-occurring at the contact zone’s centre. Inter-subtaxon hybridization at this centre and a continuum of quality habitat for *M*. *natalensis* show that both viruses have the ecological opportunity to spread into the other substaxon’s range, but do not, strongly suggesting host-intrinsic barriers. Such barriers could explain why human cases of another *M*. *natalensis*-borne arenavirus, Lassa virus, are limited to West Africa.

## Author Summary

Reservoirs of zoonotic viruses are usually equated with a particular wildlife species. It is rarely assessed whether genetic groups below the species level may instead represent the actual reservoir, though this would have major implications on estimations of the zoonosis’ spatial distribution. Here we investigate whether geographically and genetically distinct subtaxa of the widespread African rodent *Mastomys natalensis* carry distinct arenaviruses, by sampling in detail across a contact zone of two of these subtaxa. Ongoing hybridization shows that individuals of the subtaxa are in direct physical contact, in principle allowing viral exchange, yet neither of the two arenaviruses -Gairo and Morogoro virus- were found to have crossed the zone. Such intraspecific genetic barriers to arenavirus spatial spread have important implications for our understanding of the related Lassa arenavirus, a pathogen potentially lethal to humans of which *Mastomys natalensis* is also the main reservoir. Although Lassa virus appears to infect several secondary hosts, its distribution is restricted to West Africa and matches that of another *M*. *natalensis* subtaxon. Our data thus indicates that it is because of *M*. *natalensis* intraspecific distinctions that the human Lassa fever endemic area has not expanded to the rest of sub-Saharan Africa.

## Introduction

Most emerging RNA virus infections originate from wild animals [1]. Fortunately, outbreaks of many such infections are geographically restricted, e.g. MERS coronavirus in the Middle East, Marburg filovirus in central and southern Africa, and Nipah henipavirus in south-east Asia. This restriction is most likely due to dependence on particular host reservoir species (single or multiple) for persistence in nature–those hosts themselves having restricted distributions. Identification of the reservoir host and its geographic range are therefore essential for informed public health responses [2], dramatically illustrated by the 2014 outbreak of Zaire-Ebola virus that unexpectedly emerged in West Africa [3].

Repeated detection of a particular virus in a particular animal species, and not in other species in sympatry, usually implicates that species as the main reservoir. However, species with a wide geographic range are often (cryptically) genetically subdivided into subtaxa, yet it is rarely assessed whether a local intraspecific taxon may represent the reservoir instead of the entire species. Such associations between intraspecific animal taxa and particular viral taxa could explain why the distribution of some viruses appears smaller than the range of the reservoir species, for example in the case of distinct hantaviruses of the widespread rodent species *Peromyscus leucopus* [4, 5], *P*. *maniculatus* [4, 6, 7] and *Oligoryzomys flavescens* [8], and the Simian Immunodeficiency viruses (SIV) and Simian Foamy viruses (SFV) of chimpanzees (*Pan troglodytes*) [9–12]. However, it is difficult to corroborate these associations. Experimental infections require housing individuals of the particular subtaxa in biosafety laboratories, and will in any case reflect capacity of the virus to infect the host, rather than whether the host has a reservoir status in nature: whether an infection may be persistently transmitted in a particular host population depends on more factors than the ability to propagate in a host body after manual inoculation. For example, the route and timing of viral shedding in concordance with the host’s population dynamics may be important determinants of the infection’s invasion and persistence probabilities in a host population [13, 14]. Inferring subtaxon-virus associations from observations in nature may be more appropriate, but when spatial gaps or coinciding geographic barriers occur between sampling points of the distinct subtaxa, host-extrinsic factors such as isolation-by-distance or host movement barriers are indistinguishable as explanations of the spatial separation of viruses. Therefore, the association between virus and host taxa must be evaluated in areas where distinct host (sub)taxa carrying distinct viral taxa are in direct physical contact.

This situation can be found in secondary contact zones. These are formed when vicariant subtaxa that had allopatrically diverged in the past, re-expand into secondary contact during favourable environmental conditions [15]. Commonly, this contact results in the production of fertile hybrids across a delineated and stable hybrid zone [16]. These limited zones are often maintained by a balance between dispersal and (endogenous) selection against hybrids, so that distinctive genepools co-exist in the face of gene flow [17]. For example, using fine scale sampling across the European house mouse hybrid zone, it was demonstrated that strains of Murine cytomegalovirus and of the protozoan *Cryptosporidium tyzzeri* are each associated with a distinct *Mus musculus* subspecies [18, 19]. For RNA viruses, it might be expected such secondary contact zones provide the optimal ecological conditions for an evolutionary host shift to the closely related taxon across the zone, as even for host-specific RNA viruses their high mutation rates might ensure rapid adaptation to the exposed novel host. However, this has not yet been evaluated in nature. Host shift potential of RNA viruses has previously mainly been studied by comparing genealogical histories of virus phylogenetic groups and of their corresponding hosts [20–26]. This has allowed identification of e.g. phylogenetic distance between host taxa as an important constraint for a host shift; yet it remains unclear whether such a constraint may last when closely related subtaxa carrying different RNA viruses physically meet, for example at a secondary contact zone. Here, we characterize a secondary contact zone of subtaxa of the African rodent *Mastomys natalensis* to better understand host-imposed constraints to the distribution patterns of the rodent’s arenaviruses.

**Fig 1:**
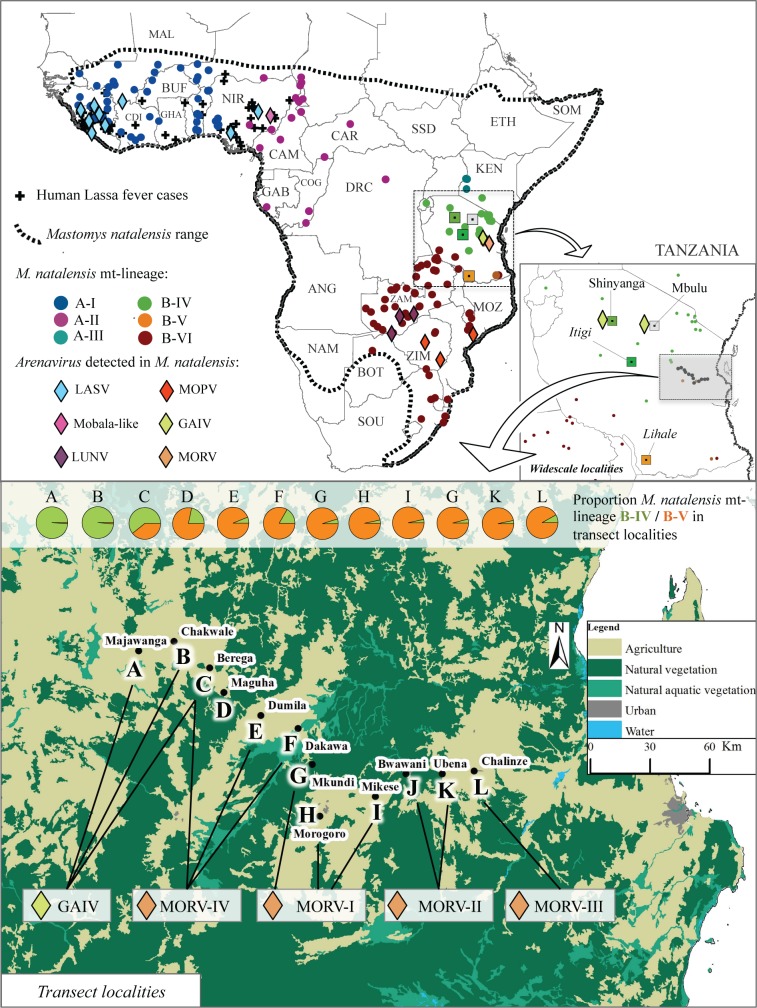
Overview of occurrences of *M*. *natalensis* and its arenaviruses and this study’s sampling localities. Top: Map of Africa, indicating georeferenced human Lassa fever cases (crosses) [40–42], occurrence of *M*. *natalensis* genetic matrilineages (circles) as determined by [43, 44] and reported occurrences of arenaviruses in *M*. *natalensis* (diamonds) [31–33, 35–39, 45]. Inset: map of Tanzania with “wide-scale” sampled localities, where squares indicate the *M*. *natalensis* matrilineage that was detected, according to the colour code of the Africa map. Grey square indicates *M*. *natalensis* could not be genotyped from that locality. Bottom: Map of sampled “transect” localities, with indication of the proportion of *M*. *natalensis* matrilineages B-IV and B-V (pie-charts) and arenavirus strains (diamonds) present in each locality.

Arenaviruses are bi-segmented RNA viruses and those of the genus *Mammarenavirus* are typically hosted by rodents. Only a few can successfully infect humans, and while human-to-human transmission is possible, it has so far never resulted in a sustained epidemic [27, 28]. Lassa mammarenavirus (LASV) may cause a severe haemorrhagic fever in humans, and with about 200,000 cases and 3,000 deaths annually [29] it has a major public health impact [30]. LASV’s main natural reservoir host species is the Natal multimammate mouse *Mastomys natalensis* [31–33], although recently LASV and LASV-related strains have also been detected in other rodent species [34]. While this common rodent occurs throughout most of sub-Saharan Africa, LASV and Lassa fever in humans is restricted to West Africa and has never been detected east of Nigeria (Fig 1). Instead, five other arenaviruses have so far been detected from *M*. *natalensis* in various other regions (Fig 1): Mopeia virus (MOPV) in Mozambique [35], Morogoro virus (MORV–a strain of MOPV) in Tanzania [36], Luna virus (LUV) in Zambia [37], Gairo virus (GAIV) in Tanzania [38] and recently an unnamed Mobala-like virus in east-Nigeria [39]. These have never been detected in humans.

Mammarenaviruses are in general considered rodent-host specific. The majority has only been detected in a single rodent species, and on several occasions distinct arenaviruses have been found in distinct rodent species captured at the same sites [45–49]. On the other hand, five arenaviruses have been detected in multiple, yet closely related, rodent species [34, 50–53], suggesting that the level of host specificity may vary between arenaviruses.

*M*. *natalensis* is one of the most widespread and common mammals in sub-Saharan Africa; it occurs in all terrestrial habitats apart from dense forests and (semi-) deserts [54, 55]. Its mitochondrial DNA can be divided into six matrilineages that differ up to 3.8% at the cytochrome b gene and that are each geographically confined to distinct regions [43]. When superimposing the distributions of these matrilineages and *M*. *natalensis*-borne arenaviruses, each arenavirus seems to be restricted to the range of a single matrilineage, the Lassa fever endemic area roughly matching the range of *M*. *natalensis* matrilineage A-I (Fig 1). This pattern suggests that host intraspecific structure, as approximated by the matrilineal pattern, may constrain the geographic ranges of arenaviruses including LASV. Recently, Olayemi *et al*. (2016) concluded this is not the case, as they detected LASV in three *M*. *natalensis* individuals carrying the A-II mitochondrial lineage in eastern Nigeria. However, no nuclear markers were typed, and the A-II mitochondrial lineage was observed in less than 25% of the animals in the two localities where these LASV positive animals were detected, located at the edge of A-I matrilineage distribution. Moreover, a different and new arenavirus was found in an eastern locality across the river Niger where the A-II mitochondrial lineage was found in all individuals. Therefore, it remains unclear whether the observed pattern was due to LASV dispersal into the range of the host taxon associated with the A-II matrilineage, or due to A-II mitochondrial introgression into the range of the host taxon carrying LASV. Indeed, introgression of mitochondrial lineages into other taxa is a common phenomenon, therefore the spatial distribution of mitochondrial lineages often does not closely match that of the (multi-locus inferred) taxa themselves [56].

In central Tanzania, *M*. *natalensis*–borne GAIV and MORV are known to occur in close proximity [36, 38], and the subtaxa potentially represented by *M*. *natalensis* matrilineages B-IV and B-V are estimated to be in secondary contact in that region [43]. We previously found within the B-V matrilineage range that nuclear markers are further substructured in relation to an urban-rural contrast, and that the region varies in landscape features that likely translate into spatially varying *M*. *natalensis* densities [57]. Such areas of low host densities and/or inter-host contacts might in themselves present a sufficient barrier for virus transmission [58], and should therefore be taken into account when distinguishing host-intrinsic and host-extrinsic factors of viral distribution patterns.

In this study, we sampled *M*. *natalensis* at a fine scale across the spatial transition zone between GAIV and MORV, multilocus genotyped hosts and their arenaviruses, and estimated landscape connectivity between localities. In the context of this natural laboratory, we are able to discern the contributions of host genetic structure and landscape features on the spatial distribution of arenaviruses.

## Methods

### Sample collection

We sampled small mammals at twelve localities along the road between Dar es Salaam and Dodoma, spaced approximately 20 km apart and spanning the distribution range boundary of the B-IV and B-V *M*. *natalensis* matrilineages [43] and Gairo and Morogoro arenaviruses (Fig 1). Part of the sampling overlaps with data presented in [38] and [57]; see Table 1. At each locality small mammals were captured in Sherman live traps baited with a mixture of peanut butter and maize flour. Traps were set in a 1 ha square grid of 10x10 traps in fallow lands. At each locality minimum two grids were constructed minimum 500 m, maximum 2.5 km apart. If trapping success was low after two nights, we set additional grids. For each sampled grid maximum 20 *M*. *natalensis* individuals were euthanized by Isoflurane inhalation.

**Table 1:** Summary of trapping and genotyping data per locality. *M*. *natalensis* trapping success, average assignment of the microsatellite genotypes to the yellow cluster at K = 2 (see Fig 2), frequency of the matrilineage B-IV, frequency of *smcy* allele A, prevalence of GAIV and prevalence of MORV in dried blood samples.

				*M*. *natalensis*				Arenavirus in blood samples	
	Locality	Latitude, longitude	Sampling time	Nr of captures / trap nights (trapping success in %)	Average percentage of microsatellite genotypes assigned to yellow cluster +- variance [N]	Mitochondrial lineage B-IV / Nr genotyped for cytochrome b (%)	*smcy* A / Nr of males genotyped for *smcy* (%)	GAIV RNA positive / nr tested (GAIV prevalence [%])	MORV RNA positive / nr tested (MORV prevalence [%])
**Transect localities**	A–Majawanga*	-6.11, 36.82	August 2012	106 / 400 (26.5)	81.6 ± 0.4 [102]	97 / 98 (99.0)	34 / 36 (94.4)	18 / 106 (17.0)	0 / 106 (0)
	B–Chakwale*	-6.05, 36.96	August 2012	84 / 200 (42.0)	98 ± 0.1 [80]	69 / 69 (100)	24 / 24 (100)	1 / 84 (1.2)	0 / 84 (0)
	C–Berega*	-6.18, 37.14	August 2012	142 / 800 (17.8)	53.5 ± 0.6 [96]	64 / 111 (57.7)	28 / 45 (62.2)	4 / 141 (2.8)	0 / 141 (0)
	C–Berega**	-6.18, 37.14	December 2009	23 / 700 (3.3)	51.3 ± 0.4 [23]	18 / 23 (78.3)	6 /12 (50.0)	0 / 23 (0)	0 / 23 (0)
	D–Maguha**	-6.29, 37.19	December 2009	23 / 800 (2.9)	17.9 ± 0.5 [23]	5 / 23 (21.7)	3 / 13 (23.1)	0 / 23 (0)	0 / 23 (0)
	E–Dumila**	-6.38, 37.36	December 2009	152 / 400 (38.0)	10.2 ± 0.4 [124]	6 / 98 (6.1)	0 / 37 (0)	0 / 152 (0)	4 / 152 (2.6)
	F–Dakawa**	-6.45, 37.54	December 2009	36 / 400 9.0)	8.3 ± 0.1 [36]	6 / 36 (16.7)	0 / 17 (0)	0 / 36 (0)	1 / 36 (2.8)
	G–Mkundi**	-6.62, 37.60	December 2009	21 / 400 (5.3)	1.6 ± 0.1 [20]	1 / 20 (5.0)	0 / 8 (0)	0 / 21 (0)	1 / 21 (4.8)
	H–Morogoro**	-6.85, 37.65	December 2009	133 / 400 (33.3)	2.4 ± 0 [71]	2 / 75 (2.7)	0 /29 (0)	0 / 133 (0)	4 / 133 (3.0)
	H–Morogoro**	-6.85, 37.65	December 2010	277 / 400 (69.3)	2.3 ± 0 [29]	1 / 17 (5.9)	0 / 6 (0)	0 / 157 (0)	4 / 157 (2.5)
	I–Mikese**	-6.77, 37.86	January 2011	98 / 500 (19.6)	7.7 ± 0.1 [33]	1 /29 (3.4)	0 / 17 (0)	0 / 98 (0)	2 / 98 (2.0)
	J–Bwawani**	-6.66, 38.03	January 2011	61 / 800 (7.6)	3.6 ± 0.1 [22]	1 / 25 (4.0)	0 / 13 (0)	0 / 61 (0)	3 / 61 (4.9)
	K–Ubena**	-6.64, 38.19	January 2011	54 / 900 (6.0)	14.9 ± 0.2 [27]	1 / 31 (3.2)	0 / 18 (0)	0 / 54 (0)	3 / 54 (5.6)
	L–Chalinze**	-6.66, 38.36	December 2010-January 2011	78 / 1100 (7.1)	6.3 +- 0.1 [35]	3 / 37 (8.1)	0 / 18 (0)	0 / 78 (0)	2 / 78 (2.6)
**Wide-scale extra localities**	Mbulu	-4.08, 35.60	January & November 2011		ND	ND	ND	2 / 47 (4.3)	0 / 47 (0)
	Shinyanga-Lubaga**	-3.64, 33.42	July-August 2009		93.6 ± 0 [7]	2 / 2 (100)	ND	1 / 4 (25)	0 / 4 (0)
	Itigi**	-5.74, 34.41	July-August 2010		92.1 ± 0.1 [16]	15 / 15 (100)	ND	0 / 10 (0)	0 / 10 (0)
	Berega (C)	-6.18, 37.14	January 2007 to February 2008		ND	ND	ND	0 / 207 (0)	1 / 207 (0.5)
	Berega (C)	-6.18, 37.14	March 2008 to February 2009		ND	1 / 2 (50)	ND	2 / 110 (1.8)	0 / 110 (0)
	Lihale**	-10.80, 35.17	July-August 2008		0.6 ± 0 [16]	0 / 15 (0)	ND	0 / 14 (0)	0 / 14 (0)

**Table legend/footnotes:** For each of the three *M*. *natalensis* markers there are only two types, therefore: percentage of the blue cluster = 100 –percentage of the yellow cluster; percentage of matrilineage B-V = 100 –percentage of B-IV; percentage *smcy* allele T = 100 –percentage allele A. ND: not determined. For sampling sessions indicated with *, rodent trapping, arenavirus and *M*. *natalensis* cytochrome b data were previously reported in [38]; for sampling sessions indicated with **, rodent trapping, *M*. *natalensis* cytochrome b and microsatellite data were previously reported in [57]. Rodent trapping for three “wide-scale extra collections”, Mbulu, Berega 2007–2008 and Berega 2008–2009, was previously reported in [59–61].

Blood was drawn either from the retro-orbital sinus or the punctured heart with a capillary tube and preserved on pre-punched filter papers (Serobuvard, LDA 22, 106 Zoopole, France), and organ samples were preserved in RNAlater and ethanol. RNAlater samples were kept at 4°C for maximum six weeks prior to storage at -80°C. When more than 20 *M*. *natalensis* were captured in a grid, supernumerary animals were sedated through Isoflurane inhalation, blood and toe-clips sampled on filter paper and in ethanol, respectively, and each was released at point of capture. Molecular screening of arenaviruses was augmented with six dried-blood sample collections from previously published rodent-trapping work [57, 59–61] (see details in Table 1). *M*. *natalensis* genotyping for microsatellite and cytochrome b markers (see below) was augmented using three of these additional collections (see Table 1).

### Ethics statement

All animal work was approved by the University of Antwerp Ethical Committee for Animal Experimentation (2011–52), and followed regulations of the Research Policy of Sokoine University of Agriculture as stipulated in the “Code of Conduct for Research Ethics” (Revised version of 2012). Euthanasia of small mammals was performed using an overdose of Isoflurane or via cervical dislocation.

### *M*. *natalensis* genotyping and characterization of genetic structure

DNA was extracted from toe or liver samples using the DNeasy Blood & Tissue Kit (Qiagen). Fifteen microsatellite loci [62] were genotyped as described in [57]. However, only those samples for which more than 10 loci were successfully amplified were considered for downstream analyses. Parts of cytochrome b (on the maternally inherited mitochondrion) and *smcy* (on the paternally inherited Y chromosome) were amplified in PCRs and Sanger sequenced in one direction. See further PCR details in in S1 Text.

We analysed the population genetic structure of *M*. *natalensis* microsatellite genotypes using the Bayesian clustering algorithm implemented in the program STRUCTURE v2.3.2. [63, 64], using the same settings as described in [57]. In brief, genetic clusters are sought in which deviation from genetic disequilibria are minimised, with proportions of each microsatellite genotype assigned to each of K clusters. The analysis was replicated 25 times for each K value, allowing for admixture and using a prior on shared sampling location (at the locality-level). Modes in STRUCTURE outputs were distinguished using CLUMPAK; similar level-K replicates are placed in the same mode, within-mode cluster labels are standardized, and assignments across modes and K levels calculated [65]. Similarity between clusters at *different* K-levels and modes was assessed by eye and for visual clarity given the same colour.

Cytochrome b sequences were aligned and compared to published *M*. *natalensis* sequences by constructing a Maximum Likelihood phylogenetic tree in RAxML (GTR substitution model, gamma rate variation, 1000 bootstraps) [66]. Each sequence was then assigned to lineage B-IV or lineage B-V as described in [43]. *Smcy* sequences were aligned in Geneious 6.1 using the Geneious alignment algorithm with a 5.0/-9.203 match/mismatch cost model.

### Arenavirus detection and genotyping

Arenavirus RNA was screened in RNA extracted from dried blood samples (pooled by two) using two independent one-step reverse transcription-PCRs (RT-PCRs) targeting the same 340 nucleotide (nt) portion of the RNA-dependent RNA polymerase gene (L segment), but with different primers with different target affinities (see details in S1 Text). For pools positive for this viral gene (and a subset of 347 negative samples), additional RNA was extracted from individual kidney biopsies preserved in RNAlater using the Nucleospin RNA II kit (Macherey-Nagel) when available. From these RNA extract parts of the GPC gene (979 nt or 234 nt) and NP gene (558 nt or 450 nt) were amplified. All amplicons were Sanger sequenced in both directions. See S1 Text for further details on these assays.

### Arenavirus phylogenetic analyses

We aligned our L, NP and GPC sequences with arenavirus sequences available in GenBank (all from rodents, except Lujo virus) in Geneious 6.1 based on the translated amino acid sequences (Blosum62 cost matrix). We removed the short non-coding parts, and constructed the phylogenetic trees of each partial gene sequence in MrBayes, (GTR substitution model, gamma rate variation: 6 categories, rate parameters estimated separately for each codon). Since we were only interested in topology and not in dating nodes, we minimised parameters by using a uniformly distributed strict clock prior on branch lengths. We let 4 MCMC chains run for 1 million iterations after the standard deviation of the split frequency reached 0.01. The replicate analyses without assuming a clock model (unconstrained branch lengths using an exponential prior probability distribution) did not significantly differ in likelihood or topology (S3 Fig).

### Correlations between spatial genetic structure and landscape patterns

As a quantitative measure of the potential for *M*. *natalensis* to move between localities, i.e. a measure of environmental barriers, we estimated landscape resistance pairwise between localities of the transect (Fig 1, localities A to L, and excluding all other sampled localities), using the methodology of [57] but applied over a larger geographic extent. In brief, ten field experts in *M*. *natalensis* ecology translated landscape elements of Tanzania’s land cover layer of 1997 [67] to exponentially increasing categories of *M*. *natalensis* habitat quality. After integrating linear landscape elements into this habitat quality layer (rivers and roads of three different width categories), the resulting expert opinion layer was modelled using Circuitscape [68] as a conductive surface from which resistance values between pairs of polygons (minimum polygons drawn around sampling sites in each locality) are calculated, in analogy with circuit theory.

The centroids of each locality and the earth (great-circle) distance between them were calculated in the R package ‘fields’ [69]. We based the mean genetic distance between arenavirus samples of each locality on a concatenation of the three arenavirus gene sequences (to a total of 1848 nucleotides) and then calculated this mean distance between localities in MEGA 5.2 (Tamura 3-parameter model) [70].

The correlation between the genetic distance matrices and the landscape resistance distance matrix or earth distance matrix was calculated using simple Mantel tests in the R package ecodist [71] (1,000,000 permutations for bootstrapping). Partial Mantel tests of the same package were used to correlate the genetic distance matrices to the landscape resistance distance matrix while ‘partialling out’ the influence of earth distance between localities.

### Characterizing the *M*. *natalensis* contact zone

Sampling coordinates were transformed to a flat surface using gnomonic projection. The centroids of the twelve transect localities (Fig 1, A to L) were then orthogonally projected onto their regression line to form a one-dimensional transect. Narrow clines are robust to such mapping details [72]. For each locality, frequencies of the *M*. *natalensis* matri- (cytochrome b) and patri- (*smcy* flank) lineages and the average assignment to either of the microsatellite clusters in the integrated Q matrix of the K = 2 STRUCTURE scenario were tabulated. Numbers of MORV and GAIV in the total arenavirus infected (RT-PCR positive) animals were tabulated per locality. We fitted clines to these observations using the software Analyse [73].

### *M*. *natalensis* relatedness (kinship)

To evaluate whether by chance we sampled more related animals in some localities than others, we calculated Li’s relationship coefficient *r* [74] between pairs of host genotypes within each locality in SPAGeDi [75]. See details in supplementary Methods in S1 Text.

## Data Archiving

Genetic sequences generated in this study are deposited in GenBank:

- 681 *M*. *natalensis* cytochrome b (accession numbers: KF779499-KF779870; KP140966-KP141215; KY283164—KY283167), excluding 45 low quality sequences for which we could still determine the mitochondrial lineage;
- 269 *M*. *natalensis smcy* (accession numbers: KY283277—KY283545), excluding 24 low quality sequences for which we could still determine the *smcy* lineage;
- arenavirus partial L gene (accession numbers: KJ856580-KJ856601, KY283168—KY283197),
- arenavirus partial NP gene (accession numbers: KJ856602-KJ856623, KY283252—KY283276),
- arenavirus partial GPC gene (accession numbers: KY283198—KY283251).

Original data deposited in the Dryad repository: http://dx.doi.org/10.5061/dryad.5n00k [76]:

- Field and laboratory data (sample locations, dates and species identification of captured animals, RT-PCR data of arenavirus screening, GenBank accession numbers)
- Microsatellite data
- Parameter settings for STRUCTURE
- MrBayes data and parameter settings input files
- Landscape costs (the costs assigned to landscape elements for connectivity analyses in Circuitscape)

## Results

### *M*. *natalensis* spatial genetic structure in central Tanzania

**Fig 2:**
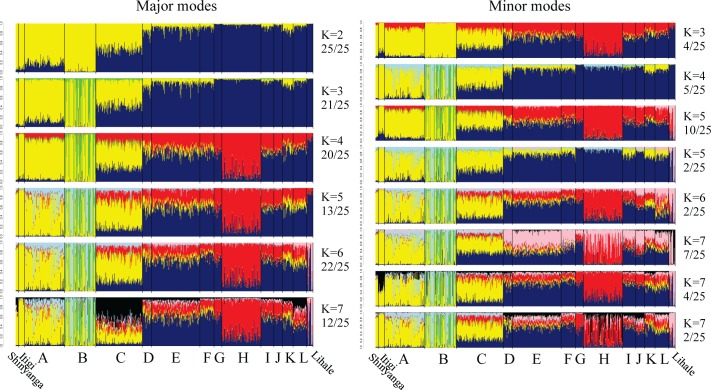
Population-genetic characterization of *M*. *natalensis* from microsatellite markers. For each microsatellite genotype, the proportional assignment to each of two to seven clusters (K) as calculated in STRUCTURE [63, 64]. Grouping of STRUCTURE runs into different modes, cross-labeling clusters per mode and per K and calculation of average cluster-assignment per mode and per K was performed in CLUMPAK [65]. Genotypes are arranged per sampling locality. Left plots indicate the modes into which the majority of 25 STRUCTURE runs could be categorized (how many is indicated on the right hand side of the plots); right plots indicate the minor modes. Similarity between clusters of different K-values and different modes was evaluated by eye, with similar clusters being depicted with the same colour.

Of 1,289 *M*. *natalensis* individuals sampled in 12 localities, a random subset was genotyped, as well as 39 additional *M*. *natalensis* individuals from one southern and two northern localities in Tanzania (“wide-scale localities”; Fig 1, Table 1). We identified two *M*. *natalensis* cytochrome b matrilineages (B-IV and B-V *sensu* Colangelo *et al*. (2013) [43]; S1 Fig), as well as a bi-allelic SNP in the Y chromosome *smcy*-gene intron, suggesting two distinct patrilineages (later referred as A and T lineages) (Table 1). When allowing for two microsatellite clusters (K = 2) in STRUCTURE, all replicate runs (25/25) converged to the same spatial pattern of genetic structure (Fig 2), strongly supporting a division of our sample into two replicable genetic disequilibrium-minimising groups. Outwith the transect, in northern localities Shinyanga and Itigi all *M*. *natalensis* carried the mitochondrial B-IV lineage and belonged to microsatellite cluster 1 (yellow), while in the southern locality Lihale all mice carried B-V mitochondrial lineage and belonged to microsatellite cluster 2 (blue) (Table 1, Fig 2).

**Fig 3:**
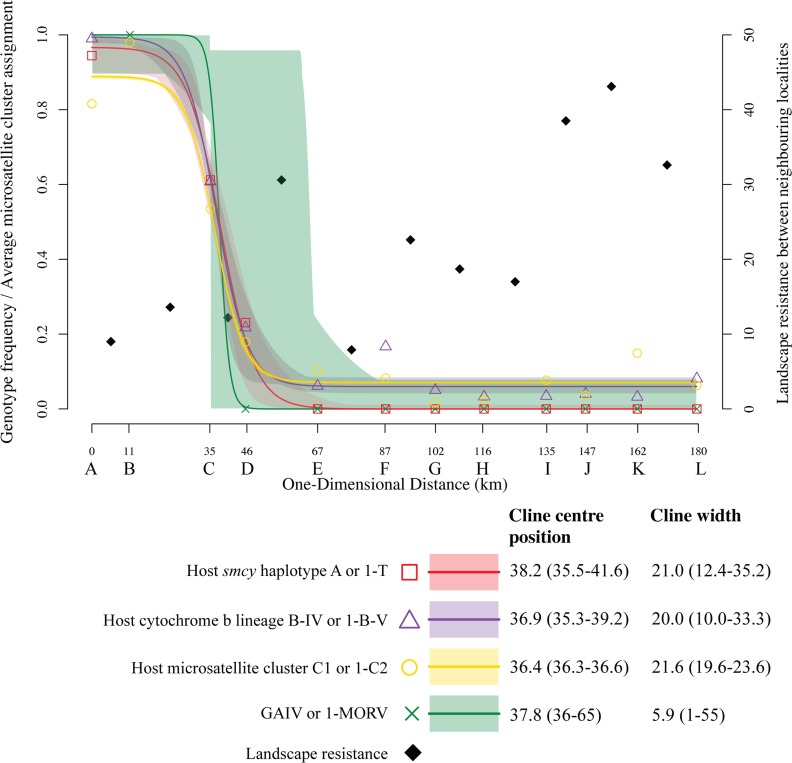
Genotype frequency clines of two *M*. *natalensis* cytochrome b and *smcy* types (binomial error distribution), clines of average assignment to two *M*. *natalensis* microsatellite clusters (from STRUCTURE’s K = 2 scenario, Gaussian error distribution) and frequencies of arenavirus species (MORV and GAIV, binomial error distribution). The one-dimensional positions of the localities (A to L) are their orthogonal projection onto the regression line fit between the centroids of the localities. The support envelopes (polygons) for expected genotype frequencies (lines) under the standard cline model were estimated in Analyse [73]. Squares, triangles, circles and crosses represent the observed proportions/frequencies. Maximum Likelihood estimates and their 2-unit support intervals for cline centres and widths are shown at the bottom right. *M*. *natalensis* estimated landscape resistance between neighbouring localities (right y-axis) is depicted with black-filled diamonds at the midpoint between locality pairs (see also S1 Table).

Along the sampled transect the proportions of the two matrilineages, patrilineages and microsatellite cluster memberships changes sharply between localities B and E (Fig 3, Table 1). In locality C, Berega, both maternal and paternal lineages are present and autosomal hybrid genotypes dominate (Table 1, Fig 2), indicating ongoing hybridization between two *M*. *natalensis* subtaxa. The clines for all three *M*. *natalensis* genomic compartments (mitochondrion, Y chromosome and autosomes) have narrow confidence intervals and very similar estimated cline centre positions (near locality C) and cline widths (Fig 3). The consistency across genomic compartments and the relatively narrow estimated cline widths (20.0, 21.0 and 21.6 km, respectively–Fig 3) indicate a multilocus barrier to gene flow between the two *M*. *natalensis* subtaxa. Together with the observations from the wide-scale localities, we can conclude *M*. *natalensis* matrilineages B-IV and B-V correspond with genome-wide genetic structure in this region. We will therefore subsequently refer to these genome-wide clusters as *M*. *natalensis* subtaxa B-IV and B-V.

The B-IV mitochondrial lineage, but not the *smcy* A allele, is observed at low frequencies throughout the sampled transect localities in B-V’s range (localities E-L, up to 140 km from the estimated clines centres), indicating wide scale but low level mitochondrial introgression (Table 1). Low level introgression of both B-V mitochondrial lineage and *smcy* T allele was also observed in the sampled transect localities in the B-IV subtaxon range (localities A and B), but this range was only sampled up to 40 km from the estimated clines centers.

For STRUCTURE K>2, the two subtaxa’s microsatellite clusters are further hierarchically substructured with consistent spatial pattern (Fig 2). At K = 3 (major modes) or K = 4 (minor modes), animals from locality B (Chakwale) form a sub-cluster embedded within subtaxon B-IV. This substructure may be due to significantly higher levels of relatedness within this locality than in others (S1 Text and S2 Fig). At K = 3 (minor modes) or K = 4 (major modes), animals from locality H (Morogoro) also form a sub-cluster embedded within subtaxon B-V, consistent with previous inference over a subset of this dataset [57]. These animals do not show high relative relatedness patterns (S1 Text and S2 Fig). At K = 5 (minor modes) and K = 6 (major modes), a subset of the animals at the southern locality Lihale are consistently distinguished. These animals are significantly more related to each other than animals in other localities (S1 Text and S2 Fig).

### Arenavirus genetic structure

**Fig 4:**
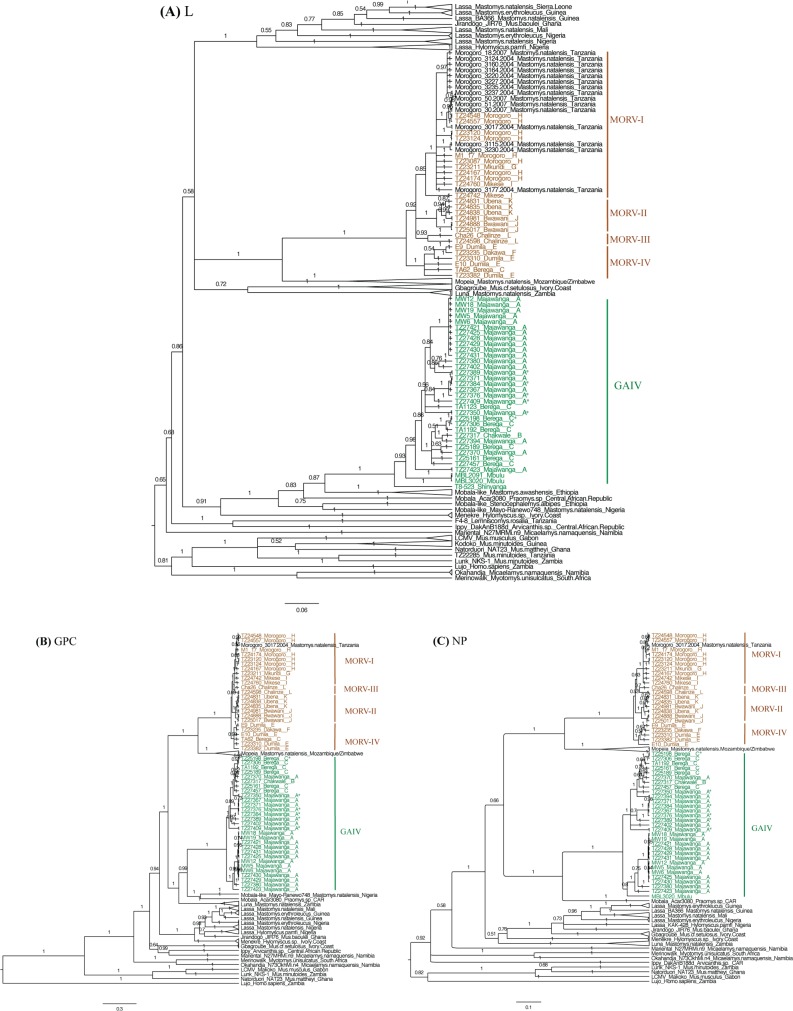
Phylogenetic trees constructed in MrBayes [77] using GTR substitution model and uniformly distributed strict clock prior on branch lengths, based on (**A**) a 340 nt partial sequence of the L gene, (**B**) up to 1512 nt partial sequence of GPC gene, and (**C**) 542 nt partial sequence of NP gene. Trees were rooted on Lujo virus. Sequences of all known rodent-borne arenaviruses (except Lujo virus which was isolated from human patients only) from Africa (in black) and those sampled in this study (in colour) are included. Numbers on branches represent posterior probabilities of the clades’ monophyly. The arenaviruses detected only from kidney samples instead of dried blood in this study are indicated with asterisks (*).

A total of 53 arenavirus positive samples were found in 1,167 dried blood samples (DBS) from the transect-localities and in 392 blood samples from additional collections (Table 1). A further 6 out of 347 kidney samples tested (from individuals with negative DBS) were arenavirus RT-PCR positive. Phylogenetic reconstruction of parts of the L, GPC and NP genes showed positive samples contained the arenaviruses MORV and GAIV (Fig 4).

Bayesian phylogenetic trees based on the partial L (Fig 4A), GPC (Fig 4B) and NP gene (Fig 4C) showed four clades for MORV that were each specific to a single or set of adjacent localities (Fig 1). One clade (MORV-III) was however not well supported by the phylogenetic tree based on NP sequences (Fig 4C). MORV-III monophyly was also not supported by phylogenetic reconstruction without branch length constraints (S3 Fig). The remaining topology was similar under clock and non-clock models (S3 Fig).

Along the transect GAIV was only detected in majority B-IV host subtaxon localities and MORV only where the B-V host subtaxon was in the majority (Table 1, Figs 1, 3 and 4). The only exception was the hybrid-host-rich locality C, Berega, where both GAIV and MORV were detected at low prevalence (1.5% and 0.2%, respectively; Table 1). GAIV was also found in Mbulu and Shinyanga in north Tanzania, consistent with it being present across the range of the northern B-IV subtaxon (Fig 1).

The spatial frequency clines of the two arenaviruses thus coincide with their host’s genotypic clines, with estimated virus cline centre and width falling within the 95% confidence intervals of those of the host clines (Fig 3), although virus cline confidence intervals are much broader due the lower number of virus observations. The association between viral type and host taxa on either side of the cline centre is highly significant (χ^2^ = 42, df = 1, p = 9.1 x 10^−11^).

### Comparing host-virus distribution to landscape structure

Both virus and host transition zones centre around locality C (Fig 3). Pairwise landscape resistance varies across the localities within the zone centre’s confidence intervals (B-C-D-E-F, 13.6, 12.2, 30.6, 7.9, Fig 3, S1 Table) with maximum between D-E. Across the transect as a whole, J-K-L comparisons have higher resistance estimates than D-E. Neither of these regions of high host-movement-resistance are likely to match the true centre of the GAIV-MORV transition zone nor appear to genetically structure the arenavirus associated with the B-V host subtaxon: MORV occurs on both sides of D-E and the clade MORV-II occurs in localities J as well as K. A partial mantel test correlating mean arenavirus (GAIV+MORV) genetic distance between pairwise localities to the landscape resistance, while partialing out the earth distance matrix, was not significant (R^2^ = -0.13, p = 0.95). Mantel tests (both simple and partial) lead to a high number of false-positive correlations [78], making this lack of correlation more striking. To summarise: away from the host contact zone landscape resistance is stronger than that within the zone, and even at its strongest, does not correlate with intraspecific arenavirus genetic substructure.

### Comparing intraspecific arenavirus structure to within-subtaxon genetic and landscape structure

In contrast to the large-scale geographic association between arenavirus species and *M*. *natalensis* subtaxa, there was no geographic match between population genetic structure of *M*. *natalensis* within the B-V taxon (Fig 2) and the sublineages of MORV (Fig 1 and Fig 4). *M*. *natalensis*-B-V from locality H (Morogoro) show genetic separation from surrounding localities, but their MORV strains are of the same sublineage as those of adjacent localities G and I (Fig 1, Fig 2, Fig 4). *M*. *natalensis* from all other B-V localities belong to the same population genetic cluster, while carrying four distinct MORV sublineages (Fig 1, Fig 2 and Fig 4). A partial mantel test correlating mean MORV genetic distance between pairwise localities (excluding GAIV specific localities A and B) to the landscape resistance, while partialling out earth distance was significant (R^2^ = 0.02, p = 0.001) but weaker than a simple mantel test correlating mean genetic distance to earth distance (R^2^ = 0.38, p<0.001), indicating isolation-by-distance. GAIV’s range was not sampled broadly enough for equivalent analyses in relation to structure within *M*. *natalensis*-B-IV subtaxon.

## Discussion

Our results demonstrate that the *Mastomys natalensis* arenavirus system in central Tanzania does indeed form a natural laboratory suited for distinguishing host-intrinsic and environmental factors limiting the spread of arenaviruses. Multilocus genotyping shows a narrow contact zone between two subtaxa of *M*. *natalensis*. The ranges of these subtaxa are largely consistent with previously described matrilineages, though low frequency widespread mitochondrial introgression is also observed. The *M*. *natalensis* subtaxa each carry a different arenavirus right into their contact zone, where clustering of nuclear genotypes reveals ongoing hybridization. This shows that members of the subtaxa are in direct individual-to-individual contact, yet neither virus is found to have crossed the zone. Landscape analyses furthermore show the largest indicators of barriers to host movement or troughs in host density in our study area lie *outside* the zone and do not correlate with interspecific or even intraspecific viral genetic substructure. However, a number of alternative hypotheses for the observed *M*. *natalensis* B-IV—GAIV and B-V—MORV associations remain to be discussed.

1) The host taxa and their viruses could have only recently arrived at their current boundaries, the arenaviruses lacking the time to spread through new host taxon’s range. Using multiple fossil calibration points, the matrilineages of the subtaxa were previously estimated to have diverged allopatrically about 1.4 million years ago, during humid climate that could have encouraged dense rainforests (a *M*. *natalensis* barrier) to surround isolated patches of savannah (suitable *M*. *natalensis* habitat) [43]. Since then the African climate has experienced many fluctuations, but after the last African Humid Period between 14,800 and 5,500 years ago, during which large parts of East Africa would have been forested, climate has not been very different from its current state [79]. It therefore seems reasonable to suggest the *M*. *natalensis* subtaxa could have come into contact any time during at least the last 5,500 years, making it unlikely the time window in which the contact zone was formed coincided with our sampling.

2) Arenaviruses of *M*. *natalensis* appear to cause mainly acute infections with subsequently life long presence of antibodies [38, 80–82]; and antibodies are widely cross-reactive between several Old-World mammarenaviruses [83, 84], which could lead to cross-immunity [85]. Such cross-immunity on first inspection might appear to have a potential role blocking viral transmission across a contact zone of two host taxa with different arenaviruses. However, on closer inspection, this appears highly unlikely for our study system. Frequency of immune individuals on either side of the host contact is clearly below any threshold allowing viral spread and persistence. Complete cross-immunity would mean a spreading virus would encounter the same frequency of immune individuals as the virus already present–which by definition is below any threshold allowing viral spread and persistence. Cross-immunity is not, therefore, the factor blocking viral spread.

3) The available landscape layers for Tanzania might not cover all possible environmental barriers for *M*. *natalensis*-borne arenavirus dispersal. In particular the host/arenavirus clines roughly correlate with the position of an altitudinal cline (S4 Fig). Local precipitation patterns vary along the altitudinal cline (S4 Fig) and are determinants of onset and duration of the *M*. *natalensis* breeding period [86]. Nevertheless, the duration and peaks of rainfall still largely overlap between localities north (Mbulu), within (Berega), and south (Morogoro) of the contact zone (S4 Fig), and *M*. *natalensis* directly monitored in Berega and Morogoro indicate that breeding seasons are not timed very differently on either side of the contact zone: while in some years *M*. *natalensis*’ breeding onset can differ north-south by 3 months, in others it is well synchronized [60, 86]. While breeding asynchrony may, in the case of cross-immunity, cause a delay in virus transmission from a high-density population (where transmission rates could be increasing due to recruitment of susceptible juveniles) to a low-density population (which consists mostly of adults that are more likely to have cross-reactive antibodies [38, 80, 81]), this delay would only be temporary and would certainly not be able to explain a long-term geographic separation of the two arenaviruses.

Therefore, our findings strongly suggest spread of the two arenaviruses in question across the *M*. *natalensis* subtaxa contact zone is blocked by host-intrinsic rather than geographic/environmental factors. We thus directly assess how two arenaviruses fail to successfully emerge into closely related host taxa, despite optimal ecological exposure conditions, highlighting the strong limits to RNA virus adaptive flexibility that host-imposed constraints may enforce. These constraints might not be present at lower levels of host differentiation than the two subtaxa: we did not observe a spatial match between intraspecific genetic structure of MORV and population genetic structure within *M*. *natalensis* B-V, where an urban population has only recently differentiated from rural mice [57].

These insights highlight how our understanding of zoonotic virus epidemiology could strongly benefit from an investment in molecular taxonomic research of wildlife reservoir hosts, and that the intraspecific level deserves more attention. Small mammals such as rodents and bats have been indicated as a major source of viral zoonoses [87], but much of the diversity that these animals harbour is likely to be cryptic and understudied at the genetic or phylogeographic level. For example, only 66% of the 2277 rodent species currently recognized worldwide have more than three DNA sequences deposited in GenBank (accessed 30/01/2016). We therefore argue that thorough taxonomic and phylogenetic investigations of reservoir hosts should go hand in hand with zoonoses surveillance programs.

### Potential host-intrinsic factors

We show evidence suggesting host-intrinsic factors determine the spatial distributions of two arenaviruses. This paves the way for experimental studies investigating what such factors might be. Candidate factors should include a wide range of possibilities from direct host immune defense differences to indirect effects, for example differences in infection-mediated behavior that enhance viral transmission. Interactions with other infections/symbionts should not be ignored: the host taxa may have, for example, diverged not only in their genomes but also in their gut microbiomes. It should also be borne in mind that host-intrinsic effects may be associated with hybrids, which can show vigorous immune response due to heterosis [88]. The combination of mechanisms involved is unlikely to be simple. For example, the configuration of α-dystroglycan, the (known) main cell entry receptor of Old-World mammarenaviruses is invariant across several species of the *Mastomys* genus [89], ruling this out as a simple explanation. Replicating relevant aspects of the population-level process of viral transmission in the laboratory will therefore be challenging.

### Implications for the estimation of LASV distribution range

As mentioned in the introduction, the human Lassa fever endemic area roughly matches the distribution range of *M*. *natalensis* A-I matrilineage, but recently, Olayemi et al. (2016) found LASV in three *M*. *natalensis* individuals carrying the A-II mitochondrion and concluded that LASV may spread to the rest of the A-II matrilineage range (which extends up to eastern Democratic Republic of Congo; Fig 1; [43]) [39]. However, the observations in Olayemi et al. (2016) actually appear consistent with the current study: 1) frequencies of matrilineages A-I and A-II (only mitochondria were typed) gradually changed along a west-east axis; 2) LASV and no other arenavirus was found in two localities where A-I predominated over A-II (1/9 and 2/9 genotyped animals carrying A-II, the remainder A-I); 3) a different, Mobala-like arenavirus, but not LASV, was found in localities where A-II mitochondrion predominates (A-II found in 3/3 genotyped animals, and lies east of a locality where A-II was found in 17/19 animals). As the authors note, *M*. *natalensis* subtaxa associated with A-I and A-II matrilineages thus likely form a hybrid zone in eastern Nigeria, potentially coinciding with the river Niger. Comparing these findings with our multilocus fine-scale data from Tanzania, we would predict that the locality where LASV was detected in three individuals with A-II mitochondria lies west of the hybrid zone’s centre, across which these A-II mitochondrial copies have introgressed, as is common in contact zones [56] and as we also observed in this study. We would thus predict multilocus (not mitochondrial) genotyping would cluster those three individuals in the “A-I subtaxon”. The convergent spatial patterns in Olayemi et al. (2016) and this study are thus consistent with a general association of the arenaviruses of *M*. *natalensis* to particular subtaxa, implying that LASV is restricted to the West African range of the subtaxon corresponding with A-I matrilineage.

However, firstly the potential dispersal barrier effect of the river Niger on *M*. *natalensis* nuclear gene flow and LASV transmission should be evaluated as alternative explanation. Secondly, the role of other rodent hosts in the spatial spread of LASV needs to be clarified. It has recently become clear that several strains of LASV may be harboured by rodent species other than *M*. *natalensis*, namely the closely related *M*. *erythroleucus* and *Hylomyscus pamfi* [34], and divergent LASV-related strains have been found in distantly related *Mus baoulei* and *Mus cf*. *setulosus* [90].

Two important questions thus remain: (1) is LASV a generalist whereas other African arenaviruses, especially GAIV and MORV, are specialists? This would explain why LASV is found in a wide array of species, including humans, but fails to explain why the distribution of LASV appears largely bordered by the distribution of *M*. *natalensis* A-I matrilineage. Therefore: (2) Do hosts other than *M*. *natalensis* A-I subtaxon contribute to the long-term persistence of LASV in nature?

*M*. *erythroleucus* has been found carrying LASV in an area just outside of *M*. *natalensis’* range (coastal Guinea) and where human Lassa fever cases have also been reported–implying that either humans or *M*. *erythroleucus* managed to import LASV and establish a transmission chain without involvement of *M*. *natalensis* [34]. On the other hand, it is clear that these importations have not (yet) occurred very far outside of the *M*. *natalensis* A-I matrilineage range, despite the continuous distribution of *M*. *erythroleucus*, *H*. *pamfi* and of course humans through to other parts of Africa. It therefore seems that either: 1) *M*. *erythroleucus* and *H*. *pamfi* are not able to sustain a long-term persistent LASV transmission, similar to the situation in humans [27, 28]. Such less efficient transmission could e.g. be due to differences in intra-host infection dynamics and population ecology of these species in comparison to *M*. *natalensis*. 2) LASV is sustainably transmitted in *M*. *erythroleucus* and *H*. *pamfi* populations, and LASV’s distribution in reality, and unnoticed, expands throughout the ranges of *M*. *erythroleucus* and *H*. *pamfi* (though note that at least *M*. *erythroleucus* is known to be subdivided into subtaxa similar to *M*. *natalensis* [91]). Perhaps they carry LASV strains less pathogenic to humans elsewhere; such a situation has previously been postulated to explain the absence of reported Lassa fever patients in regions in Mali where a particular LASV strain has only recently been found to be common in *M*. *natalensis* [32]. 3) LASV strains in *M*. *erythroleucus* and *H*. *pamfi* are the result of adaptive host switching events that occurred only recently, and have yet to expand through the rest of the rodents’ ranges.

It is clear more surveillance of arenaviruses in rodents, especially in the area bordering the Lassa fever endemic area, is needed to fully answer the pressing question: should we expect LASV and its associated diseases to emerge in the rest of Africa?

## Supporting Information

S1 Text Supplementary information on material and methods.Genotyping mitochondrial and *smcy* markers.Arenavirus screening and genotyping.Table A. Overview and details of different arenavirus screening and genotyping RT-PCR assays used in this study.Supplementary Results.Prevalences per locality.*M*. *natalensis* relatedness (kinship).
(DOCX)

S1 Fig Maximum-likelihood phylogenetic tree based on cytochrome b sequences of *Mastomys natalensis* sampled in this study and in Colangelo et al. (2013) [43]. (PDF)

S2 Fig Average (circles) and standard error (error bars) of Li’s relatedness coefficient between pairs of *M*. *natalensis* individuals from each locality. (PDF)

S3 Fig Phylogenetic trees constructed in MrBayes [77] using GTR substitution model with unconstrained branch lengths, based on (**A**) a 340 nt partial sequence of the L gene, (**B**) up to 1512 nt partial sequence of GPC gene, and (**C**) 542 nt partial sequence of NP gene. Trees were rooted on Lujo virus. Sequences of all known rodent-borne arenaviruses (except Lujo virus which was isolated from human patients only) from Africa (in black) and those sampled in this study (in colour) are included. Numbers on branches represent posterior probabilities of the clades’ monophyly. The arenaviruses detected only from kidney samples instead of dried blood in this study are indicated with asterisks (*).
(PDF)

S4 Fig Seasonal precipitation patterns across Tanzania. Base layer is relative altitude. The average monthly precipitation for selected weather stations is depicted in the graphs. Sampled localities are indicated by red dots, black dots represent the weather stations from which the precipitation data was derived. The numbers next to the black dots indicate the month of the year with the highest precipitation (1: January, 2: February, etc.).
(PDF)

S1 Table Landscape resistance (in terms of the reciprocal of *M*. *natalensis* habitat quality) between each pair of transect localities (see main text Fig 1). In boldface are pairwise values between neighbouring localities, which are also depicted in Fig 3 in the main text.
(XLSX)
