# Supplementary material for: When Viruses Don’t Go Viral: The Importance of Host Phylogeographic Structure in the Spatial Spread of Arenaviruses
Source: PLoS Pathog. 2017 Jan 11;13(1):e1006073. doi: 10.1371/journal.ppat.1006073 (PMC5226678; doi:10.1371/journal.ppat.1006073)
Supplement: S1 Text — Supplementary information on material and methods.Genotyping mitochondrial and smcy markers.Arenavirus screening and genotyping.Table A. Overview and details of different arenavirus screening and genotyping RT-PCR assays used in this study.Supplementary Results.Prevalences per locality.M. natalensis relatedness (kinship). (DOCX) [file ppat.1006073.s001.docx]

**S1 Text**

1. Supplementary Methodology

1. 1. Genotyping mitochondrial and smcy markers

Parts of cytochrome b (on the maternally inherited mitochondrion) and *smcy* (on the paternally inherited Y chromosome) were amplified in PCRs containing 0.2 µM of each dNTP (Fermentas), 0.2 µM of each primer, and either 1X DreamTaq Buffer + 1.25 U DreamTaq DNA Polymerase (Thermo Fisher Scientific Inc.) or 1X GoTaq® Flexi Buffer and 1.25U GoTaq® DNA Polymerase (Promega). Cytochrome b primers, H15915 and L14723, targeted a 1140 bp region [1]. *Smcy* primers, smcy_28F (5'- TATCCACTGTWAATGAAACTWTGC-3´) and smcy_24R (5'-TATCTTGCAGTTACGTCACAT-3'), were designed based on primer sequences from [2] and *Mus musculus*, *Hylomyscus alleni* and *Mastomys huberti* (*hildebrandtii*) *smcy* sequences in GenBank [3] and targeted a 924 bp region across an *smcy* exon and intron. All amplicons were Sanger-sequenced in one direction using respectively primers L14723 and smcy_24R at the Genetic Service Facility (GSF) of the Vlaams Instituut voor Biotechnologie (VIB, Antwerp, Belgium).

1.2. Arenavirus screening and genotyping

Viral RNA was extracted from dried blood samples, pooled by two, using the methodology described in [4]. These RNA extracts were screened for presence of arenavirus RNA with two independent one-step reverse transcription-PCRs (RT-PCRs) that target the same 340 nucleotide (nt) portion of the RNA-dependent RNA polymerase gene (L segment), but using different primers with different target affinities (see details on MoroL and Pan-arenavirusL assays in Table A below). RT-PCRs were performed with the SuperScript One-Step Platinum Taq RT-PCR System (Invitrogen), with 1x Reaction Mix, 625 µM MgSO_4_, 0.4 µL enzyme mix, and primers in 0.4 µM (LVL_3359D_Y+ and LVL_3359G_Y+) or 0.6 µM (others) concentrations. Cycling conditions for both assays were: 30 min at 50°C; 2 min at 95°C; 45 cycles of 20 s at 95 °C, 30 s at 55°C, 1 min at 72°C; and 10 min at 72°C.

However, for 31 samples from the extra dataset of Berega, and the 28 samples of the extra datasets of Shinyanga-Lubaga, Itigi and Lihale (See Table 1 in Main Text), a different though similar screening protocol was used: RNA was extracted with the RTP® DNA/ RNA Virus Mini Kit (Invisorb, Stratec) according to manufacturers instructions (choosing the maximum lysis incubation time). RNA (eight µL of the RNA extract) was then reverse-transcribed to cDNA with Maxima Reverse Transcriptase and Random Hexamer primers (Thermo Scientific) according to manufacturers instructions. Two PCRs were then performed using 0.02 U Phusion DNA polymerase (Finnzymes, New England Biolabs), 0.2 µM dNTPs (Thermo Scientific), 0.2 µM (total) of primers, 43% of ddH_2_O and 13% of cDNA template in a total volume of 15 µL; using respectively pan-arenavirus L (in 0.4µM and 0.6µM) and NP3 primer pairs (in 0.5µM). See primer details in Table A below. Cycling conditions for both PCRs were: 1 cycle at 98°C for 30 seconds; 45 cycles of 98°C for 5 seconds, 51°C for 30 seconds, and 72°C for 20 seconds; followed by a final 72°C for 5 minutes.

PCR amplicons were bidirectionally Sanger-sequenced at the GSF-VIB to confirm arenavirus presence.

For animals from 34 positive blood sample pools, as well as a subset of 347 negative specimens, RNA was extracted from individual kidney biopsies preserved in RNAlater using the Nucleospin RNA II kit (Macherey-Nagel). For the remaining 38 individuals from 19 positive dried blood sample (DBS) pools, organ samples in RNAlater were not available therefore the pools were resolved by re-extracting the individual blood samples.

We then performed a one-step RT-PCR targeting either the first 1000 nt of the viral S segment (including 979 nt of the GPC gene) using primers OWS0001-fwd and OWS1000-rev [5] or, in cases where this PCR failed, a 234 nt portion of the GPC gene plus 21 nt of its flanking region using primers S36 and LVS-339-d [6]. Finally the NP gene was targeted using either a 1:1 mixture of primers OWS2805+ and OWS2810+ and a 1:1 mixture of OWS3400- and OWS3400A- [5] that targets a 519 nt of the NP gene plus 39 nt of its flanking region, or if the previous PCR failed, primers NP-A and NP-X targeting a 450 nt region within the previous NP region. See primer details in Table A below. All amplicons were bidirectionally Sanger-sequenced at the GSF-VIB.

**Table A**. Overview and details of different arenavirus screening and genotyping RT-PCR assays used in this study.

|  | RT-polymerase gene: screening a total of 1559 dried blood samples (DBS) | |
| --- | --- | --- |
| Screening Assay | Panarenavirus L | Moro L |
| Forward primer | LVL_3359D_Y+  5’-AGAATCAGTGAAAGGGAAAGCAAYTC-3’  LVL_3359G_Y+  5’-AGAATTAGTGAAAGGGAGAGTAAYTC-3’ | MoroL3359+  5’-AGGATTAGTGAGAGAGAGAGTAATTC-3’ |
| Reverse primer | LVL_3754A_R-  5’-CACATCATTGGTCCCCATTTACTATGRTC-3’  LVL_3754D_R-  5’-CACATCATTGGTCCCCATTTACTGTGRTC-3’ | MoroL3753-  5’-ACATCATTGGGCCCCACTTACTATGGTC-3’ |
| Target | 340 nt of RT-polymerase gene | 340 nt of RT-polymerase gene |
| Assay reference | [7] | [8] |
| DBS positive for GAIV / nr tested | 28 / 1541 (an additional 6 / 347 kidney samples –of DBS negative individuals- were positive for GAIV) | 9 / 1485 (17 samples positive with PanarenavirusL and 57 negative samples –see above- were not tested with this assay) |
| DBS positive for MORV/nr tested | 5 / 1541 (15 samples positive and 3 samples negative with MoroL were not tested) | 25 / 1485 (see remark above) |
|  |  |  |
|  | Genotyping GPC | |
| Genotyping Assay | GPC long | GPC short |
| Forward primer | OWS0001+  5'-GCGCACCGGGGATCCTAGGC-3' | FwdS36  5'-ACCGGGGATCCTAGGCATTT-3´ |
| Reverse primer | OWS1000-  5'-AGCATGTCACAAAAYTCYTCATCATG-3' | Moro_S_339rev  5'-GTTCTTTGAGCAAGAGAGAGGCATTGTTGC-3' |
| Target | ~ 1000 nt of GPC and ~ 25 nt of upstream flank (exact length dependent on arenavirus species) | 234 nt of MORV GPC and 21 nt of upstream flank |
| Assay reference | [9] | [6] |
| Genotyped / nr arenavirus positive | 45 / 53 | 3 / 53 |
|  |  |  |
|  | Genotyping NP | |
| Genotyping Assay | NP3 | NP moro |
| Forward primer | OWS2805+  5’-GTCAGGCTTGGCATTGTCCCAAACTGRTTRTT-3’  OWS2810+  5’-CTTGGCATTGTCCCAAACTGRTTRTT-3’ | MoroNP-A  5'-GGAGGTGAARTCTTTCCTTTGGA-3' |
| Reverse primer | OWS3400-  5’-GCGCACAGTGGATCCTAGGC-3’  OWS3400A-  5’-CGCAGAGTGGATCCTAGGCTATTKGATTGCGC-3’ | MoroNP-X  5'-GGGTTTYTAACATCCCAGATTC-3' |
| Target | 519 nt of NP gene and 39 nt of upstream flank | 450 nt of MORV NP |
| Assay reference | [5] | in house |
| Genotyped / nr arenavirus positive | 28 / 53 | 20 / 53 |

2. Supplementary results

2.1. Prevalences per locality

To estimate whether arenavirus prevalences were significantly different between locality C (where host subtaxon hybrids dominated) and the other transect localities (A to B and D to L), we constructed a generalised linear model with a binomial response distribution in R package lme4 [10], where locality, reproductive status and body weight of the animals were fixed effects, as these latter have previously been linked to arenavirus infection probability [11]. For this test we only used DBS from animals captured for this study from the transect localities, as this field work was performed in a uniform fashion in all localities and blood samples were genetically screened at similar time intervals after sampling.

There was an overall significant difference in arenavirus prevalence between localities (χ^2^=37.803, p<0.001). Specifically comparing with the prevalence in locality C though (where *M. natalensis* subtaxon hybrids are dominant and both MORV and GAIV were detected), only the prevalence at locality A (Z=3.715, p<0.001) was significantly higher. Note that the prevalence in locality C in this test is based on GAIV prevalence only, because the only MORV sample from this locality was detected from an animal that was not captured and genetically screened in the standardized fashion of this study but in the framework of another study (see Table 1 main text). Also, localities C and H were sampled twice at two distinct time periods. Therefore, the prevalence at these localities likely better represents the average prevalence over time than any other locality, whose prevalence estimations are thus more prone to stochasticity.

2.2. *M. natalensis* relatedness (kinship)

To evaluate whether by chance we sampled more related animals in some localities than others, we calculated Li’s relationship coefficient *r* [12] between pairs of host genotypes within each locality in SPAGeDi [13]; *r* is a summation over all loci of the average proportion of alleles shared by a pair of individuals at a given locus, including a correction for sample size and a weighting of each locus. Deviation (+,-) from 0 means that individuals within a given sample group are respectively more or less related than individuals sampled at random.

The average relatedness between individual pairs was highest in the “wide-scale” localities Shinyanga, Itigi and Lihale, which had relatively small sample sizes (see Table 1 main text), and in locality B, with a large sample (S2 Fig). Genetic structure analyses in STRUCTURE and CLUMPAK showed that some *M. natalensis* samples from locality B were significantly structured into subpopulations (see Figure 2 main text) in the absence of an environmental variable that could explain this process. It is thus not unlikely that the bias of sampling more related individuals in locality B than in other localities caused the apparent population-genetic structure within locality B.

References

1. Lecompte É, Granjon L, Peterhans JK, Denys C. Cytochrome b-based phylogeny of the Praomys group (Rodentia, Murinae): a new African radiation? C R Biol. 2002;325(7):827-40. doi: <http://dx.doi.org/10.1016/S1631-0691(02)01488-9>.

2. Sandstedt SA, Tucker PK. Evolutionary strata on the mouse X chromosome correspond to strata on the human X chromosome. Genome Res. 2004;14(2):267-72. doi: Doi 10.1101/Gr.1796204. PubMed PMID: ISI:000188811800007.

3. Sandstedt SA, Tucker PK. Male-driven evolution in closely related species of the mouse genus Mus. Journal of molecular evolution. 2005;61(1):138-44. doi: 10.1007/s00239-004-0279-1. PubMed PMID: 16007492.

4. Goüy de Bellocq J, Borremans B, Katakweba A, Makundi R, Baird SJ, Becker-Ziaja B, et al. Sympatric occurrence of 3 arenaviruses, Tanzania. Emerg Infect Dis. 2010;16(4):692-5. Epub 2010/03/31. doi: 10.3201/eid1604.091721. PubMed PMID: 20350390.

5. Ehichioya DU, Hass M, Becker-Ziaja B, Ehimuan J, Asogun DA, Fichet-Calvet E, et al. Current molecular epidemiology of Lassa virus in Nigeria. J Clin Microbiol. 2011;49(3):1157-61. Epub 2010/12/31. doi: 10.1128/JCM.01891-10. PubMed PMID: 21191050; PubMed Central PMCID: PMC3067713.

6. Ölschläger S, Lelke M, Emmerich P, Panning M, Drosten C, Hass M, et al. Improved detection of Lassa virus by reverse transcription-PCR targeting the 5′ region of S RNA. J Clin Microbiol. 2010;48(6):2009-13. doi: 10.1128/JCM.02351-09. PubMed PMID: PMC2884523.

7. Vieth S, Drosten C, Lenz O, Vincent M, Omilabu S, Hass M, et al. RT-PCR assay for detection of Lassa virus and related Old World arenaviruses targeting the L gene. Trans R Soc Trop Med Hyg. 2007;101(12):1253-64. Epub 2007/10/02. doi: 10.1016/j.trstmh.2005.03.018. PubMed PMID: 17905372.

8. Günther S, Hoofd G, Charrel R, Roser C, Becker-Ziaja B, Lloyd G, et al. Mopeia virus-related arenavirus in Natal multimammate mice, Morogoro, Tanzania. Emerg Infect Dis. 2009;15(12):2008-12. Epub 2009/12/08. doi: 10.3201/eid1512.090864. PubMed PMID: 19961688; PubMed Central PMCID: PMC3044542.

9. Coulibaly-N'Golo D, Allali B, Kouassi SK, Fichet-Calvet E, Becker-Ziaja B, Rieger T, et al. Novel arenavirus sequences in *Hylomyscus sp.* and *Mus* (*Nannomys*) *setulosus* from Cote d'Ivoire: implications for evolution of arenaviruses in Africa. PLoS One. 2011;6(6). doi: 10.1371/journal.pone.0020893. PubMed PMID: WOS:000291612900035.

10. Bates D, Maechler M. lme4: Linear mixed-effects models using Eigen and S4. In: 1.0-5 Rpv, editor. 2013.

11. Borremans B, Leirs H, Gryseels S, Günther S, Makundi R, Goüy de Bellocq J. Presence of Mopeia Virus, an African arenavirus, related to biotope and individual rodent host characteristics; implications for virus transmission. Vector-Borne Zoonot Dis. 2011;11(8):1125-31. doi: 10.1089/vbz.2010.0010. PubMed PMID: 21142956.

12. Li CC, Weeks DE, Chakravarti A. Similarity of DNA fingerprints due to chance and relatedness. Hum Hered. 1993;43(1):45-52. doi: Doi 10.1159/000154113. PubMed PMID: ISI:A1993KR38200008.

13. Hardy OJ, Vekemans X. SPAGEDi: a versatile computer program to analyse spatial genetic structure at the individual or population levels. Mol Ecol Notes. 2002;2(4):618-20. PubMed PMID: ISI:000179678400078.
