# Supplementary figures and images for: When Viruses Don’t Go Viral: The Importance of Host Phylogeographic Structure in the Spatial Spread of Arenaviruses
Source: PLoS Pathog. 2017 Jan 11;13(1):e1006073. doi: 10.1371/journal.ppat.1006073 (PMC5226678; doi:10.1371/journal.ppat.1006073)

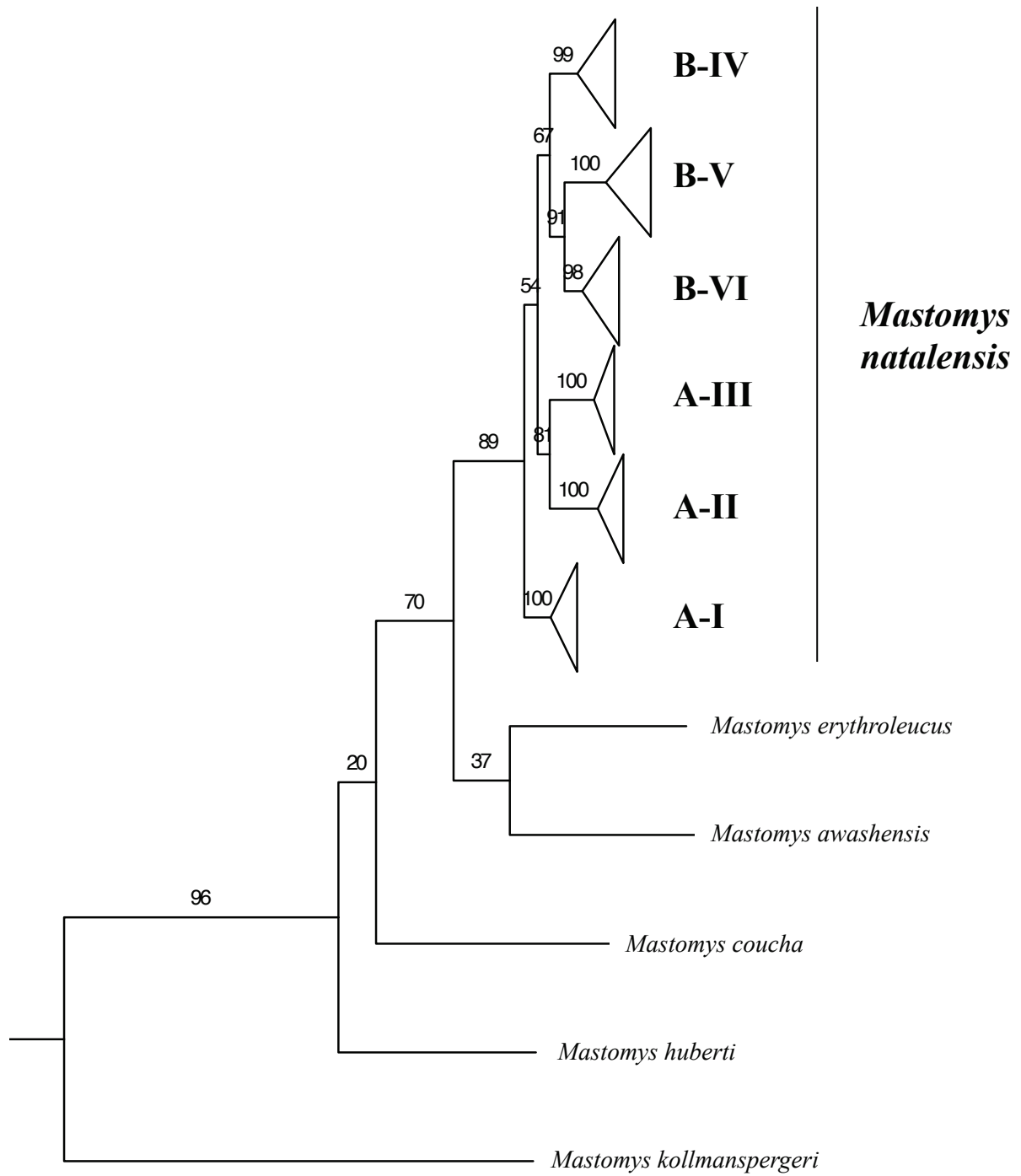

Supplement: S1 Fig — (PDF) [file ppat.1006073.s002.pdf]

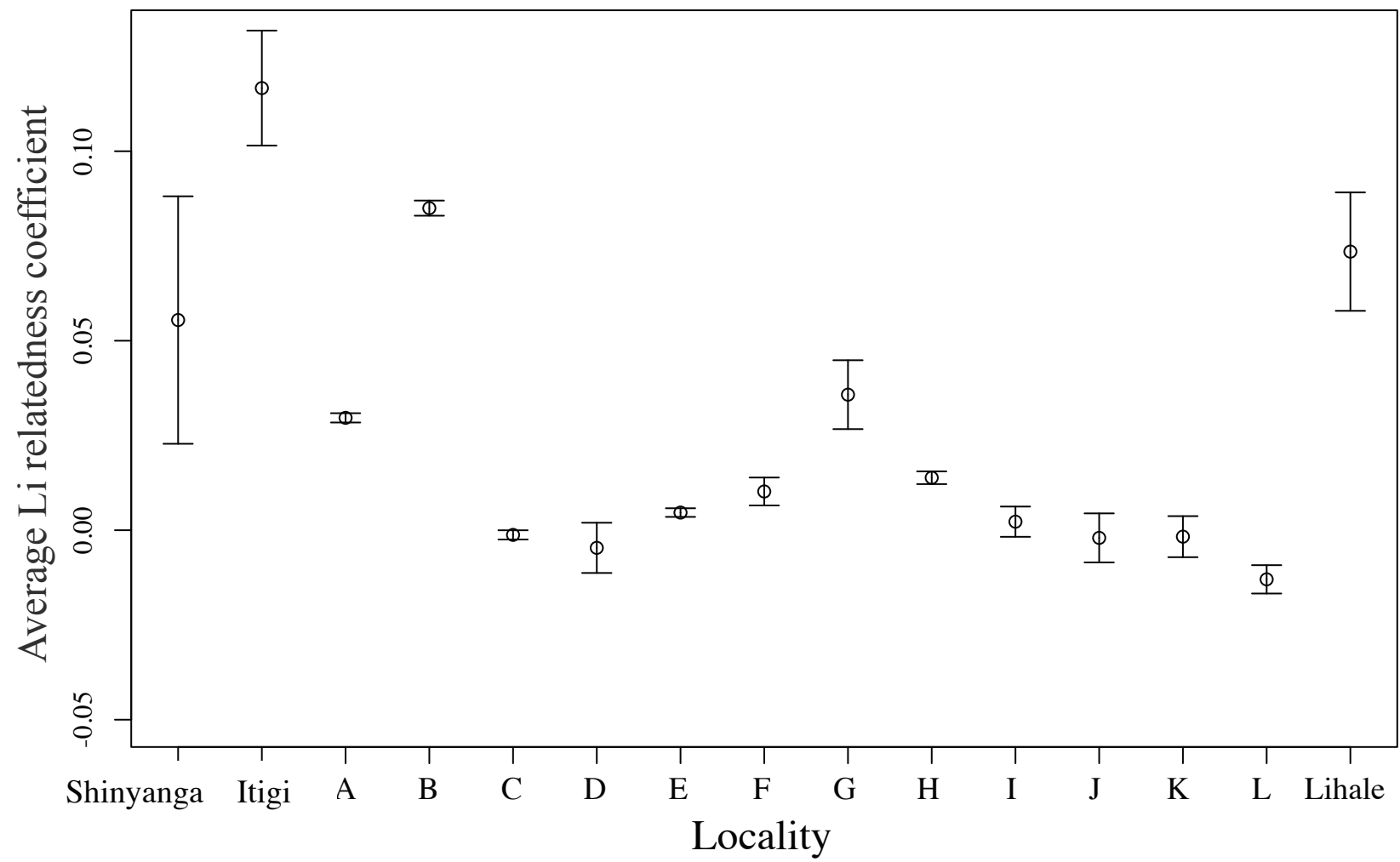

Supplement: S2 Fig — (PDF) [file ppat.1006073.s003.pdf]

(A) L

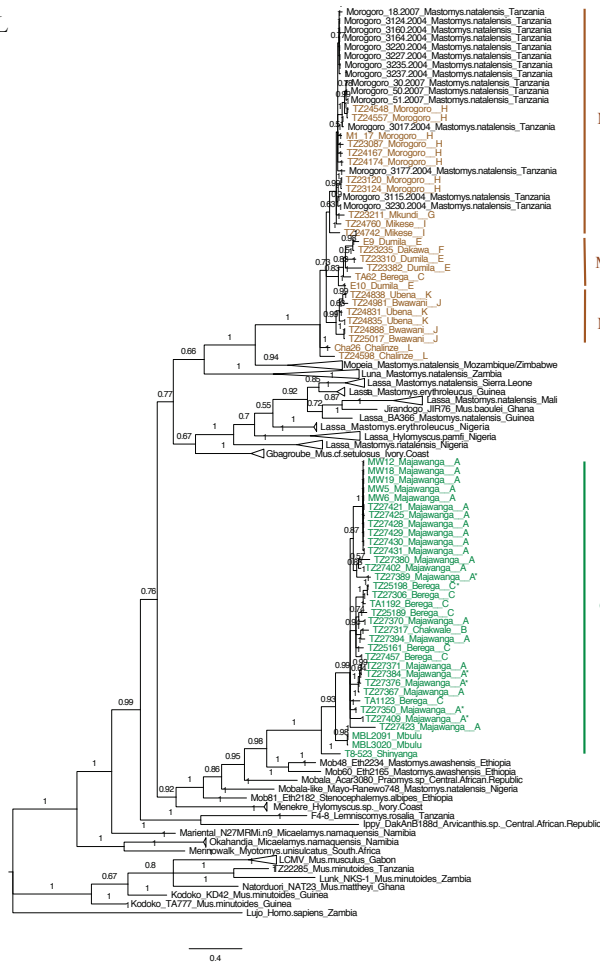

(B) GPC

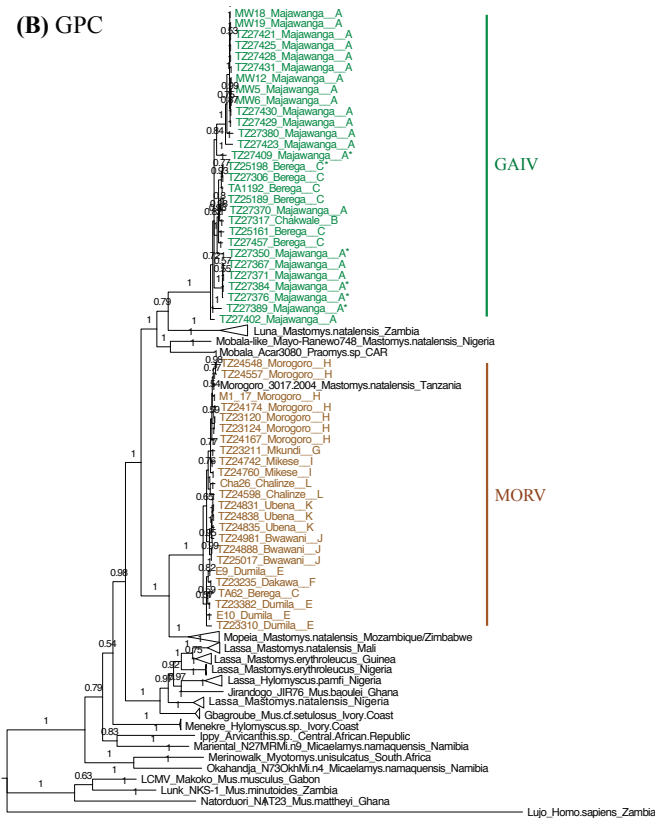

(C) NP

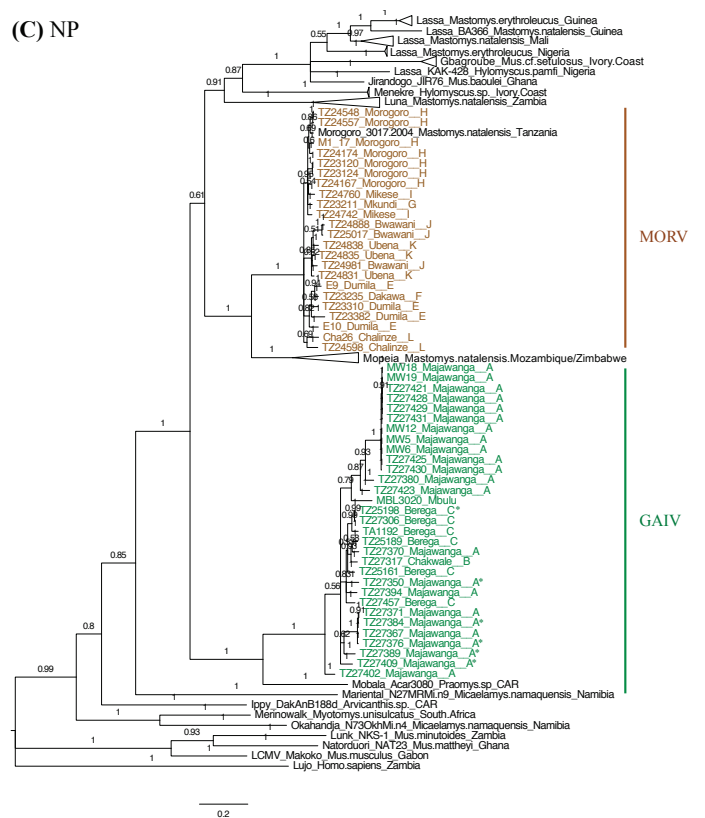

Supplement: S3 Fig — Phylogenetic trees constructed in MrBayes [77] using GTR substitution model with unconstrained branch lengths, based on (A) a 340 nt partial sequence of the L gene, (B) up to 1512 nt partial sequence of GPC gene, and (C) 542 nt partial sequence of NP gene. Trees were rooted on Lujo virus. Sequences of all known rodent-borne arenaviruses (except Lujo virus which was isolated from human patients only) from Africa (in black) and those sampled in this study (in colour) are included. Numbers on branches represent posterior probabilities of the clades’ monophyly. The arenaviruses detected only from kidney samples instead of dried blood in this study are indicated with asterisks (*). (PDF) [file ppat.1006073.s004.pdf]

Shinyanga

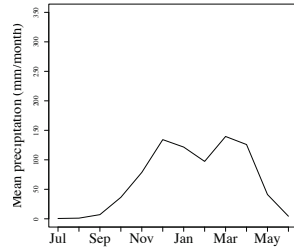

Mbulu

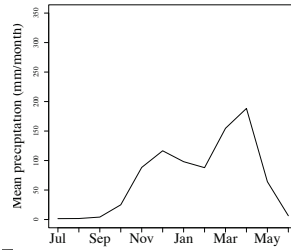

Berega

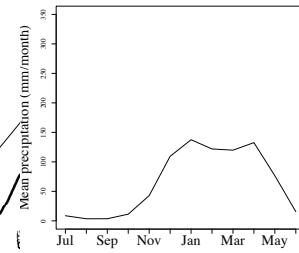

Manyoni

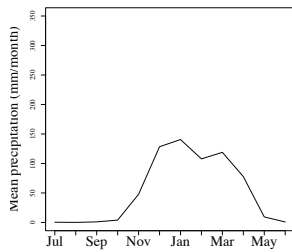

Dodoma

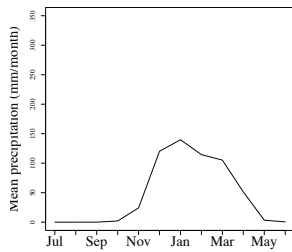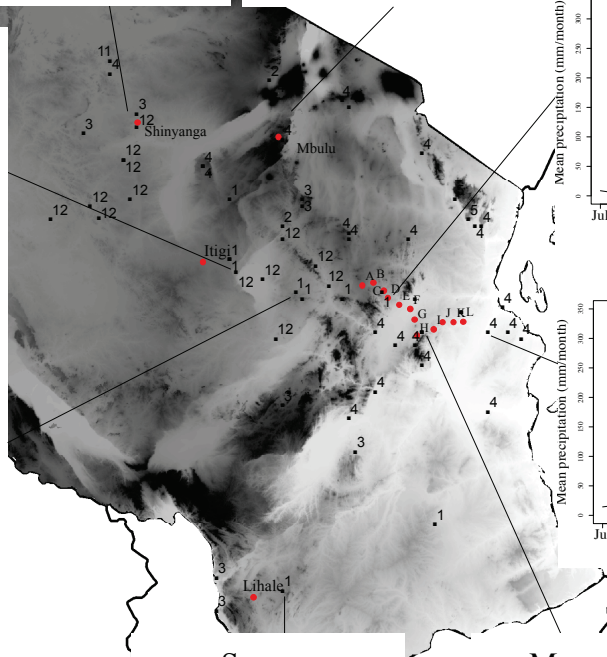

Ruvu

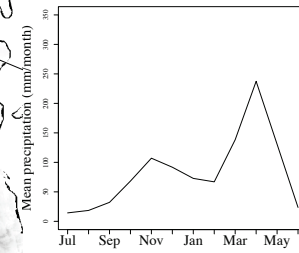

Songea

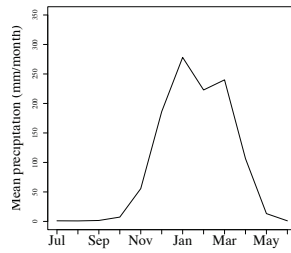

Morogoro

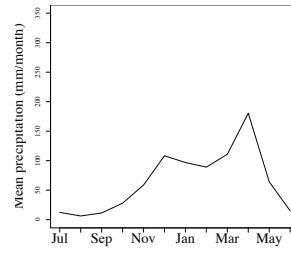

Supplement: S4 Fig — Base layer is relative altitude. The average monthly precipitation for selected weather stations is depicted in the graphs. Sampled localities are indicated by red dots, black dots represent the weather stations from which the precipitation data was derived. The numbers next to the black dots indicate the month of the year with the highest precipitation (1: January, 2: February, etc.). (PDF) [file ppat.1006073.s005.pdf]
